# Sytemic lupus erythematosus presenting with protein losing enteropathy in a resource limited centre: a case report

**DOI:** 10.1186/1755-7682-5-1

**Published:** 2012-01-26

**Authors:** Eranda C Ratnayake, Ahamed AA Riyaaz, Bandula C Wijesiriwardena

**Affiliations:** 1 Wards 45 and 46 A, National Hospital of Sri Lanka, Regent Street, Colombo, Sri Lanka

## Abstract

**Introduction:**

Systemic lupus erythematosus is a disease which may initially present with varying symptoms, most commonly a photosensitive rash and arthritis. Protein losing enteropathy is a recognized but rare presenting manifestation. Diagnosing protein losing enteropathy in resource limited centres is challenging but possible through the exclusion of other possible causes of hypoalbunaemia.

**Case Presentation:**

We report a case of protein losing gastroenteropathy secondary to intestinal lymphangiectasia as the initial manifestation of systemic lupus erythematosus in a 57 year old Sri Lankan (South Asian) male patient. The diagnosis was made by the exclusion of other causes of hypoalbuminaemia as the gold standard investigations for protein losing enteropathy were not available at this centre.

**Conclusions:**

Protein losing enteropathy is a diagnosis of exclusion in resource limited centres in the world. Systemic lupus erythematosus should be considered in the differential diagnosis of protein losing enteropathy. Intestinal lymphangiectasia should also be recognized as a possible pathophysiological mechanism.

## Introduction

Protein-losing enteropathy (PLE) is a condition characterized by hypoalbuminaemia secondary to excessive loss of serum protein from the gastrointestinal tract. Possible mechanisms of intestinal protein loss include partial lymphatic obstruction (intestinal lymphangiectasia), congenital defects in intestinal lymphatics, mucosal ulceration, disordered mucosal cell metabolism and increased central venous pressure [1].

PLE is a well recognized but unusual manifestation of systemic lupus erythematosus (SLE) [2,3]. It could rarely be the initial manifestation of the disease [4]. The exact pathogenesis of PLE in SLE remains uncertain. Intestinal or mesenteric vasculitis is a possible mechanism [5] but is rarely found in mucosal biopsies. Other hypothesized mechanisms include intestinal lymphangiectasia, mucosal disruption and increased mucosal capillary permeability due to complement- or cytokine-mediated damage [6,7]

## Case Presentation

**Figure 1:**
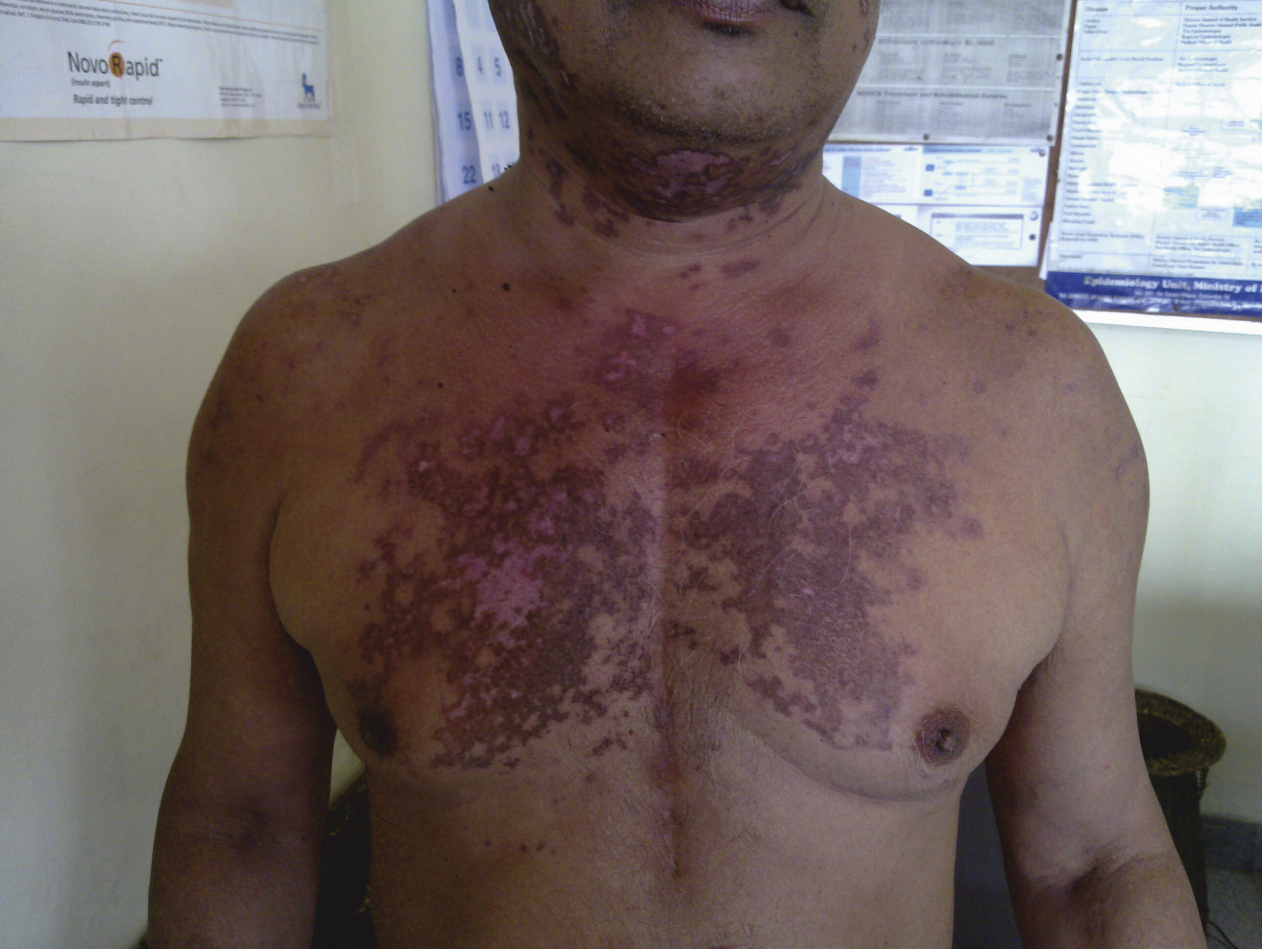
**Discoid rash on patient's neck and chest**.

**Figure 2:**
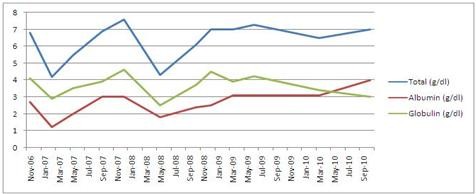
**Serum protein timeline depicting rise of serum albumin following treatment with steroids commenced in March 2010**.

A 56-year-old Sri Lankan male patient presented an year ago, with a photosensitive skin rash involving his face and upper trunk and inflammatory type symmetrical polyarthritis involving small and large joints of 3 months duration. On examination there were multiple maculo-papular cutaneous lesions with hyperpigmented borders and hypopigmented central region with scarring distributed over the face, upper chest and upper back (Figure 1). Laboratory investigations revealed a normal complete blood count and urinalysis. His ESR was 110 mm/1^st ^hour while C-reactive protein (CRP) was within normal range. Biopsy of his of his skin lesions showed an atrophic epidermis with focal basal cell degeneration, follicular plugging and scanty lymphocytic infiltration of dermis favoring a diagnosis of lupus erythematosus. Immunological investigations showed a positive ANA (titre > 1:400) and anti-ds-DNA as well reduced levels of C3 and C4. VDRL was positive but subsequent TPHA was negative. Antiphospholipid antibodies were negative. A diagnosis of Systemic Lupus Erythematosus (SLE) was made based on demonstration of 5 American Rheumatological Association criteria [8] and he was started on oral prednisolone 30 mg daily and hydroxychloroquine 200 mg twice daily. He responded to the treatment over the next few weeks as evident by the rise in serum albumin subsequently (Figure 2) along with healing of the skin rash and reduced oedema.

3 years prior to his current admission he was investigated extensively for generalized oedema, of 6 months duration and found to have severe hypoalbuminaemia without evidence of any renal or hepatic cause. He did not have significant diarrhea or steatorrhea but complained of vague left sided abdominal discomfort. Physical examination was unremarkable except for facial and mild bilateral pedal oedema. Laboratory investigations at that time were as follows: haemoglobin, 12.1 g/dl; white cell count 6.5 k/mm3 with normal differential count; platelet count 235 k/mm3; urinalysis, trace of albumin with no active sediments; 24 hour urinary protein excretion 0.12 g; serum creatinine 82 micromol/l; serum albumin 19 g/l; serum globulin 38 g/l; AST 28 u/l; ALT 30 u/l; ALP; ESR 115 mm/1^st ^hour; CRP 17 mg/l; serum total cholesterol 277 mg/dl, triglyceride 157 mg/dl, LDL cholesterol 196 mg/dl, HDL cholesterol 47 mg/dl; serum immunoglobulin assay was low normal for IgG, IgM and IgA. Ultrasonic scan of abdomen showed normal liver architecture and no evidence of portal hypertension. Percutaneous liver biopsy was histologically unremarkable. 2D echocardiography showed normal cardiac chambers and function. Barium studies of small bowel, upper and lower gastrointestinal endoscopies did not show significant pathology. Duodenal and distal ileal biopsies were done and showed evidence of lymphangiectasia without evidence of significant inflammatory cell infiltrate. A technetium labeled lymphangioscintigraphy of both lower limbs and abdomen did not show any lymphatic obstruction. A diagnosis of primary lymphangiectasia causing protein losing enteropathy was made and he was symptomatically treated with diuretics for facial and pedal oedema. Six months later, he had a non ST elevation myocardial infarction complicated by a supra ventricular tachycardia. As coronary angiography at that stage showed evidence of extensive triple vessel disease he subsequently underwent Coronary artery bypass grafting. He did not have vascular risk factors except for dyslipidaemia such as diabetes mellitus, hypertension or family history of premature atherosclerosis and was a non-smoker. He was followed up regularly and had a stable health until his current presentation.

## Discussion

Although an association between systemic lupus erythematosus and protein losing enteropathy has been recognized, there have been less than less than 150 cases documented in world literature [9]. There are no documented cases of such an association in Sri Lankan literature. Systemic lupus erythematosus *presenting *as protein losing enteropathy has been reported even less commonly.

Multiple theories as to the pathophysiology of the association between the two entities exist, such as intestinal vasculitis, mucosal erosions or lymphangiectasia, but each case seems to have its separate mechanism of protein loss. We discovered that the mechanism in our patient is through intestinal lymphangiectasia, as has been published in 2 other cases in world literature [10].

The diagnosis of protein-losing enteropathy can be successfully made by radioisotope studies or 24-hour stool _1_-antitrypsin clearance. Unfortunately these 2 investigations were not at our disposal at the National Hospital of Sri Lanka to make a concrete diagnosis. We however could confidently conclude that PLE was responsible for the hypoalbuminaema as all other causes such as urinary loss, underproduction due to liver disease and malnutrition were excluded. The association between SLE and PLE was derived mainly from the response of serum albumin following steroid treatment since March 2010 as shown in Figure 2. Other differentials for PLE such as Whipple's Disease (Non-suggestive duodenal biopsy), Crohn's disease (Negative clinical history and duodenal biopsy) and Primary Lymphangiectasia (Dramatic response to steroids) were excluded.

At the time of publishing, we have planned on performing a repeat endoscopy and re-biopsy to identify the histological response to treatment. Chase et al in 1982, were the first to postulate that intestinal lymphangiectasia in SLE was steroid responsive [11] as was shown in our case 18 years later. It is worthwhile noting that endoscopy is usually unhelpful in the diagnosis, as 50% of SLE-related PLE patients present with nonspecific intestinal wall edema and 10% of patients have no abnormalities under endoscopy examination [9]. The expected histological finding are lymphangiectasia, edematous villi and non-specific inflammation, but it can also be normal because of the inaccessibility of the involved area [1].

Although PLE associated with intestinal lymphangiectasia is associated with other phenomena such as lymphopaenia and hypolipidaemia apart from hypoalbuminaemia, it was not shown in our patient. The very high ESR with relatively normal CRP is quite characteristic of SLE without serositis [12].

It is also interesting to note the considerable lag period between his initial presentation with PLE which was followed 3 years later with the characteristic discoid rash of SLE. It is worth mentioning that SLE was not considered as a possible diagnosis at the initial stage as he did not fulfill the necessary criteria. We do not routinely perform ANA testing of patients with high ESR at our institution.

It would be natural to assume that the acute coronary syndrome suffered by the patient might be due thrombo-embolism secondary to SLE with possible secondary anti-phospholipid syndrome. This theory was dispelled however from the coronary angiography finding of extensive atherosclerotic disease.

A recent systemic review by Al-Mogairen [13] describes over a hundred patients with SLE and PLE of whom the majority of Asian in origin. Unfortunately he does not specify the Asians to be from either South or South-East Asian background. SLE with associated PLE is quite rare in the Indian Subcontinent which has a population of over 1.5 billion as evident by the limited number of published case reports.

## Conclusion

Protein losing enteropathy is a diagnosis of exclusion in resource limited centres in the world. Systemic lupus erythematosus should be considered in the differential diagnosis of protein losing enteropathy. Intestinal lymphangiectasia should also be recognized as a possible pathophysiological mechanism. The fact that this phenomenon is steroid sensitive in most instances such as in our case should be a relief to both clinicians and patients alike. The limited prevalence of this association in a highly populated territory such as the Indian Sub-Continent makes this case report intriguing.

## Consent

Written informed consent was obtained from the patient for publication of this case report and any accompanying images. A copy of the written consent is available for review by the Editor-in-Chief of the *International Archives of Medicine*.

## Competing interests

The authors declare that they have no competing interests.

## Authors' contributions

RC carried out the literature search and drafted the manuscript; WB did the critical revision for important intellectual content in the manuscript and given the final approval of the version to be published; RA helped substantially in literature search and drafting the manuscript. All authors read and approved the final manuscript.

## Acknowledgements

The authors thank the departments of gastroenterology and dermatology and histopathology of the National Hospital of Sri Lanka for providing help in the diagnosis and management of the patient described in the case report.
